# 1825. Project ECHO HIV: Engaging Clinicians to Address Substance Abuse, Interpersonal Violence, and Depression in Patients with HIV

**DOI:** 10.1093/ofid/ofad500.1654

**Published:** 2023-11-27

**Authors:** Dean K Beals, Stan Pogroszewski, Jean Anderson, Alison Livingston

**Affiliations:** DKBmed, New York, New York; DKBmed, New York, New York; Johns Hopkins University, Baltimore, Maryland; Johns Hopkins University, Baltimore, Maryland

## Abstract

**Background:**

**Finding the Invisible Patient (FIP):** Substance abuse (SA), interpersonal violence (IPV), depression and HIV are part of overlapping epidemics adversely affect HIV and the burden of each (SAVA). The Johns Hopkins HIV Women's Clinic (JHWC) initiated screening for SAVA to better identify these problems, link to needed services and evaluate the impact. Challenges included time and impact on clinic flow; need for clinic wide training and sensitization and a systems approach. **SAVA Project ECHO- FIP** took the learnings from FIP and expanded it to ten small groups across the US in order to enhance the reach and share the learnings

**Methods:**

**FIP:** Education and a series of clinic-wide changes were implemented at JHWC. Patients were now routinely screened for SAVA. and if positive sent for care. Resources were developed for patients including linkage to local assistance programs, temporary housing, posters on bathroom doors and emergency contacts. **SAVA Project ECHO- FIP** featured expert specialists to provide ongoing telementoring to clinicians through regularly scheduled educational sessions over five weeks. There were ten separate workshops, each covering a 5-part curriculum.

**Results:**

**FIP:** Of 116 women 60% were + for SAVA. Six months post-intervention, 56% were engaged in care; 39% were given a referral for psych/SUD/IPA therapy; 50% entered psychologic therapy; 28% entered substance abuse treatment; 39% for mental health issues. **SAVA Project ECHO- FIP** trained 208 clinicians to become experts in screening, identifying and treating SAVA. There was a 105% increase in confidence on identifying and treating SAVA and a 177% improvement in competence. Clinicians shared insights around building relationships with their patients and utilizing local community-based programs to help patients overcome SAVA.

**Conclusion:**

SAVA is common among many women in HIV care and is likely underestimated. There was a significant correlation with + screens and higher no show rates, reflecting increased resources needed to optimize VLS screening for components of SAVA. Screening for SAVA should be routine in HIV care settings. Resources for appropriate referral are critical. **SAVA Project ECHO- FIP** demonstrated knowledge transfer and practice improvement through its curriculum and small-group engagement.

**Disclosures:**

**Jean Anderson, MD**, Merck & Co: Stocks/Bonds

